# Evolution and clinical application of microsurgery

**DOI:** 10.1186/1753-6561-9-S3-A53

**Published:** 2015-05-19

**Authors:** Clara Wong Wing-Yee

**Affiliations:** 1 Department of Orthopaedics, Prince of Wales Hospital, Hong Kong

## 

Microsurgery is a general term for surgery requiring an operating microscope. Microscope is a word coming from two ancient Greek words, μικρός meaning “small”, and σκοπέω meaning “to see”. It is an instrument used to see objects that are too small for the naked eye. The first microscope was claimed to be invented in 1590 by Zacharias Janssen, a spectacle-maker, who tried to find a way to make magnification even greater to help people with seriously poor eyesight.

The first operating microscope, a monocular microscope, was built in 1921 in the University of Stockholm by Carl-Olof Siggesson Nylén, a Swedish otolaryngologist. He was the first to use the microscope to perfom surgeries initially on animals. In November 1921, he used the microscope to operate on the first patient, with a labyrinthine fistula. The binocular microscope that we use now was developed in 1922, by Carl’s colleague Gunnar Holmgren.

The most obvious developments in microsurgery have been procedures allowing anastomosis of successively smaller blood vessels and nerves which have allowed transfer of tissue from one part of the body to another and reattachment of severed parts. Before the term “microsurgery” appeared, vascular anastomosis was performed since 1896, well beyond the birth of operating microscope.

John Benjamin Murphy, an American physician, successfully anastomosed a femoral artery severed by gunshot wound in 1897. In 1902, Alexis Carrel, a French surgeon, originated the method of triangulation for arterial and venous anastomosis. In 1908, he devised methods for transplantation of whole organs. In 1912, he was awarded the Nobel Prize in Physiology or Medicine, for pioneering vascular anastomosis techniques and in recognition of his work on organ transplantation.

The Second World War catalysed the advancement of vascular surgery. Establishment of antibiotics, improvement in infection control, and development of fine sutures and instruments improved the results of vascular surgery and resulted in successful anastomosis of small vessels >3mm. The first surgery using microscope to aid in vascular anastomosis was done by Jules Jacobson, a vascular surgeon of the University of Vermont, who described the anastomosis of vessels as small as 1.4mm in 1960, and first used the term “microsurgery”.

The first replantation was done in 1962, by a team of chief residents led by Ronald Malt at Massachusetts General Hospital in Boston. It was a replantation at the level of the proximal humerus in a 12 year old child. But a microscope was not used at that time. The first reported replantation was done in 1963 by Chen Zhongwei in Shanghai 6th People’s Hospital in China. It was a distal forearm replantation in a mechanist. The first revascularization of a finger, a partially amputated thumb, was done in 1963, by Harold Kleinert of the University of Louisville Hand Clinic.

The first replantation of finger, a completely amputated thumb, was done in 1965, by Shigeo Komatsu and Susumu Tamai of the Medical University of Nara Prefecture. In 1966, Yang Dongyue and Gu Yudong in Huashan Hospital, Fudan University, China, did the first toe-hand transfer (2nd toe to thumb transfer). He then reported the first free groin flap in 1973. The first clinically successful free bone graft (fibula) with microvascular anastomosis was reported by Taylor In 1975. Chen Zhongwei reported on a patient with Volkman’s contracture who underwent a free pectoralis major transfer for reconstruction of flexor muscles in 1975.

With the huge number of injury cases, especially in the countries with rapid industrial growth, microsurgery became increasingly important. In the field of hand surgery, the scope of microsurgery extended from just vascular repair, to restoration of severed parts (replantation), to tissue transfer to compensate defects and to improve functions.

Microsurgery generally means doing surgery under microscope. When the microscope has to be prepared for a hand operation, people would instantly perceive that the operation is labour intensive, demands long hours and patience, and is a tiring and hard work. With the advancement of technology and bioengineering medicine, many surgeons may put microsurgery at the bottom of their treatment options exhausting many other treatment alternatives despite more expensive costs and less effective results. However, this should not be the real world of microsurgery.

Microsurgery is a concept, meaning “wide”, “deep” and “fine”. It treats a wide variety of diseases, such as defects resulted from post-traumatic, post-infective, tumour excision and congenital conditions. And, in fact, microsurgeries are performed widely in many specialties including spine, ophthalmology, gynaecology, ENT, neurosurgery, maxillofacial surgery, plastic surgery etc. With the magnified view under the microscope, a different world is seen. There is a broader and deeper understanding of a condition and disease. Microsurgery gives us a wider field of view instead of a pinhole vision. It helps us to form a more accurate diagnosis, and provides us a better platform, under the microscope, to precisely treat a disease. Arthroscopy is an example of the concept of microsurgery. With a magnified field, a clearer pathology is seen and treated under the magnified environment.

Microsurgery also represents “deep”. It does not only replenish the wound defect, but also structures deeply, including bone, joint, muscle, tendon, as a composite transfer and as a functional reconstruction. With improving technology, more advanced microscopes, sophisticated instruments and suture materials, many technical problems related to microsurgery are solved. Much finer microsurgery can be done. Distal replantation, especially in children, is an example. The distal part, previously discarded, can now be preserved, and the best quality of tissue is restored to the finger with good hand function, sensibility, psychological well-being and cosmesis.

Microsurgery represents “fine”. Finer and smaller caliber of structures are repaired. Higher cosmetic results are aimed. Donor sites are more respected. Koshima introduced perforator flaps in microsurgery, which renders the surgery less invasive, and can be more suitable for the recipient sites as they provide higher flexibility and choices for donor tissue. Supermicrosurgery enables unique operations that seemed impossible earlier, such as true perforator flaps, lymphaticovenular anastomosis. Telemicrosurgery, the installation of robot in microsurgery, makes complex microsurgeries feasible, such as brachial plexus surgery.

We should not see the general meaning of microsurgery. It is a concept, embracing wide, deep and fine. It allows to magnify, to see more, to do more, do better and help more. Until we can invent a medication which can be ingested and reforms an absent part, microsurgery will remain a precious mainstay of treatment.

